# Supporting our survivors: an evaluation of the facilitators and barriers to advocacy in cervical and breast cancer survivors

**DOI:** 10.1093/oncolo/oyaf130

**Published:** 2025-07-22

**Authors:** Lia Bos, Elizabeth Pinto, Norma Longinos, Peter Ketch, Dillon Cockrell, Teresa Boitano, Isabel Scarinci

**Affiliations:** University of Alabama at Birmingham, Division of Gynecologic Oncology, Birmingham, AL, United States; University of Alabama at Birmingham, School of Medicine, Birmingham, AL, United States; University of Alabama at Birmingham, Department of Obstetrics and Gynecology, Birmingham, AL, United States; University of Alabama at Birmingham, Division of Gynecologic Oncology, Birmingham, AL, United States; Duke University, Division of Hematology and Oncology, Raleigh, NC, United States; University of Alabama at Birmingham, Division of Gynecologic Oncology, Birmingham, AL, United States; University of Alabama at Birmingham, Department of Obstetrics and Gynecology, Birmingham, AL, United States

**Keywords:** advocacy, survivorship, cervical cancer, breast cancer

## Abstract

**Purpose:**

This study aimed to evaluate the facilitators and barriers to self-advocacy and advocacy in the community of cervical and breast cancer survivors.

**Methods:**

A mixed-methods approach was used to collect data from patients with breast and cervical cancer. Qualitative interviews were conducted after presenting for routine oncologic follow-up appointments All interviews were transcribed and coded by two separate investigators. The Female Self-Advocacy in Cancer Survivorship Scale (FSACS) was administered and demographic data were collected to quantitatively evaluate self-advocacy behaviors and socioeconomic characteristics in the two groups.

**Results:**

Twenty-one patients were interviewed: 11 cervical cancer (CC) patients and 10 breast cancer (BC) patients. The median age was 47 years, with approximately half having early-stage disease and half having advanced or recurrent disease. The facilitators to self-advocacy included being open with their support system, wanting more information, and being determined to fight. Barriers to self-advocacy included fear, not opening up to family and friends, and not wanting additional information. Facilitators to advocacy in the community were talking with their children about their diagnosis, having an interest in support groups, and a desire to help. Barriers to community advocacy included not always feeling supported, a lack of confidence, and a lack of understanding of their disease process. CC participants scored lower on the FSACS survey compared to BC patients, with mean scores of 95 and 107, respectively.

**Conclusions:**

Facilitators to advocacy involve having a strong social network and a determination to fight or desire to help. Barriers include fear, lack of confidence, and lack of knowledge. Increasing resources to encourage social connection, education, and advocacy opportunities can positively impact cancer survivors well-being.

**Implications for practice**

As the number of cancer survivors continues to grow, our healthcare system must continue to develop ways to support them and improve their quality of life. Evaluating facilitators and barriers to advocacy can help develop systematic approaches to improving cancer survivor’s participation in self-advocacy behaviors and advocacy in the community, ultimately improving the lives of survivors and those in the community.

## Introduction

Advocacy by cancer survivors has been shown to not only improve their personal quality of life but also hold the potential for widespread community benefits.^1-5^ Advocacy can take place in two main forms, community advocacy and self-advocacy. Community advocacy refers to an individual speaking out, utilizing a variety of platforms to share their story and encourage individual and systemic change. Many cancer survivors participate in advocacy in their communities by speaking with other survivors, forming support groups, assisting others in navigation of the healthcare system, encouraging screening and follow-up, and forming a united and national voice.^6^ Cancer survivors have long been considered key stakeholders in individual and community efforts for cancer screening and treatment.^7-9^ Self-advocacy refers to an individual asserting their own needs and interests in an effort to achieve their specific goals. Making informed decisions, communicating openly with healthcare professionals, and receiving support from others have been identified as skills associated with self-advocacy.^10^ Self-advocacy is becoming increasingly important as the healthcare system continues to gain complexity, and cancer patients are expected to tackle multifaceted medical decisions and care planning.^11^ Behaviors associated with advocacy have been shown to decrease symptom burden and improve the quality of life of cancer survivors.^2,3^ The idea of advocacy at both the individual and community levels has been supported by survivorship care models as a component of comprehensive care for cancer survivors.^4,5^

Despite the data supporting advocacy behaviors and education for cancer survivors, there are no studies evaluating the drivers of advocacy in patients with cancer. Breast cancer (BC) survivors are excellent examples of a group classically known for their community advocacy. Many are familiar with the pink ribbon representing breast cancer awareness, as well as publicized events to raise money for breast cancer advocacy.^12^ On the other hand, cervical cancer (CC) survivors have minimal visibility within the community. Cervical cancer survivors have also been found to have worse quality of life than both general oncologic patients and other gynecologic cancer patients.^13^ Additionally, there is limited data regarding the connection between self-advocacy behaviors and community advocacy efforts among cancer survivors. We extrapolate that these separate ideals may ultimately be connected, each with the potential to improve the quality of life of cancer survivors and community health goals. This study seeks to evaluate the facilitators and barriers of community advocacy and self-advocacy in two different patient populations with varying levels of involvement in advocacy.

## Methods

The collection and analysis of this data were focused on the facilitators of self-advocacy, the barriers of self-advocacy, the facilitators of community advocacy, and the barriers of community advocacy. Facilitators were ideas and feelings likely to encourage patients to participate in those advocacy behaviors while barriers were things perceived to inhibit or discourage those behaviors. A mixed-methods approach was used to evaluate the complexity of these drivers while providing objective characterization and evaluating the correlation with an established self-advocacy tool.

### Setting

This study was conducted at a tertiary care center with a comprehensive cancer center with patients from the gynecologic oncology department, medical oncology clinic, and breast cancer survivorship clinic. This study was approved by the institutional review board.

### Sample

Eleven CC survivors and ten BC survivors participated in a qualitative interview. Participants must have been older than 18 years of age and had completed primary treatment of their cancer within the past five years. Patients who continued on maintenance chemotherapy or who had been on the same chemotherapy regimen for at least one year were also eligible. All participants were approached during outpatient follow-up visits between March and May 2023. The interviews were completed within one visit. All eligible patients in the participating clinics were approached during this time period, enrollment ended when saturation was met. Saturation was met when no new information was being presented and themes continued to be repeated.^14^ All participants were compensated $30 for their time.

### Qualitative data

Qualitative interviews were completed in person and in the patient’s primary language (English or Spanish) by one of two trained research team members using a standardized topic guide (Supplementary 1). The topic guide consisted of open-ended questions and was organized into two parts. The first focused on patients’ experiences with their cancer diagnosis, support system, and coping strategies. The second half focused on advocacy and included questions regarding the patients’ understanding of advocacy and possible experiences as advocates. All interviews were audio-recorded for transcription. The topic guide sought to provide a framework to explore each patient’s personal motivators, personality factors, system experiences, and personal experiences that may contribute to their participation in advocacy.

All interviews were audio-recorded and subsequently transcribed. Two interviews were independently read by three separate reviewers to identify emerging themes and develop a preliminary coding schema to ensure reliable and organized coding between reviewers. This schema organized the data into sections including the patient’s experience with cancer, understanding and feelings towards advocacy, experience with advocacy, experiences in the community, thoughts about the healthcare system, barriers to advocacy, suggestions for system improvement, and many more. The remainder of the interviews were then reviewed and coded by two independent team members and compared together to ensure cohesiveness. New codes were added with each transcript and the schema was continually reviewed and refined by the two investigators. The same organization structure was used for all interviews. The resulting schema consisted of 92 themes. Major themes were determined to be any concept brought up by at least 50% of the interviewed participants.

### Quantitative data

Following the completion of the interview, the interviewer facilitated the Female Self-Advocacy in Cancer Survivorship Scale (FSACS), a 20-item survey to quantitatively assess self-advocacy skills and behaviors. The survey is divided into three sections to separately evaluate informed decision-making, effective communication, and connected strength. FSACS has been previously validated in female cancer survivors with breast or gynecologic malignancies. The highest total score is 120 with higher scores being associated with decreased symptom severity and improved quality of life.^2^

A retrospective review of the medical records of each patient was also conducted to collect sociodemographic information and information regarding their cancer diagnosis, stage, and treatment history. The quantitative data was used to better characterize the participants in the study and provide a quantitative metric for advocacy skills to compare to the qualitative information that was gathered.

## Results

### Demographics

A total of 21 patients were included in this study. The median age was 47 years, and approximately half of the patients had early-stage disease and half had advanced-stage or recurrent disease (**Supplementary Table S1**). A greater proportion of breast cancer patients had graduated from college than patients with cervical cancer (60% and 27%, respectively). The majority of patients were White (71%), 19% were Black, and 10% were Hispanic. Approximately 70% of the patients underwent chemotherapy, 70% received radiation, and 70% underwent surgery, with many undergoing multiple treatment modalities. Patient demographics of the two groups are shown in **Table 1**.

**Table 1.** Demographic information of patients.

		Breast N (%)	Cervix N (%)	Total N (%)
**Age (median)**		50	41	47
**Stage**				
	Early	5 (50)	6 (55)	11 (52)
	Advanced	5 (50)	5 (45)	10 (48)
**Education**				
	Some High School	1 (10)	4 (36)	5 (24)
	High School	0	1 (9)	1 (5)
	Some College	3 (30)	2 (18)	5 (24)
	College	4 (40)	1 (9)	5 (24)
	Graduate School	2 (20)	2 (18)	4 (19)
	Prefer not to answer		1 (9)	1 (5)
**Race**				
	Caucasian	8 (80)	7 (64)	15 (71)
	Black	2 (20)	2 (18)	4 (19)
	Hispanic		2 (18)	2 (10)
**Treatment**				
	Surgery	9 (90)	6 (55)	15 (71)
	Chemotherapy	9 (90)	5 (45)	14 (67)
	Radiation	8 (80)	7 (64)	15 (71)

### Female self-advocacy in cancer survivorship scale

The overall mean score for all 20 items was 100.9 out of a possible 120. The mean score for informed decision-making was 36.7 out of a possible 42. The mean scores for effective communication and connected strength were 30.2/36 and 34/42, respectively. Participants with breast cancer and ages over 45 had higher scores than those with cervical cancer or ages under 45. **Table 2** further describes patient scores according to age, disease stage, race/ethnicity, and treatment received by the patient.

**Table 2.** Results of the female self advocacy in cancer survivorship scale. The total score possible 120.

		Informed decision making	Effecting communication	Connected strength	Total FSACS	P-value
	All	36.67	30.19	34	100.86	
**Cancer Type**						.0175*
	Cervix (*N* = 11)	34.64	28.82	31.45	94.91	
	Breast (*N* = 10)	38.9	31.7	36.8	107.4	
**Stage**						.7724
	Early	36	30.1	35.55	101.63	
	Advanced	37.4	30.3	32.3	100	
**Age**						.0364*
	≤45	34.4	28.6	32	95	
	>45	38.73	31.6	35.82	106.18	
**Race**						
	Black or Hispanic	36.8	28.2	33.8	98.8	
	White	36.63	30.81	34.06	101.5	
**Therapy**						
	Chemo	37.43	30.79	33.79	102	
	Radiation	37.25	29.63	33.88	100.75	
	Surgery	37.47	31.13	35.6	104.2	

#### Facilitators in self-advocacy

A complete table of the major themes elicited in the qualitative interviews is found in **Table 3**. The major facilitators to self-advocacy were knowing something was wrong, being open with family and friends, relying on a strong support network, trusting their providers, being determined to fight, wanting more information, feeling connected to others who had cancer, having friends and family advocate for them, and believing self-advocacy was important (**Table 4**). Almost all were reported equally in both the CC and BC groups with the exception being determined to fight which was reported by 6 CC participants and all 10 BC participants.

**Table 3.** Major themes discussed as facilitatory and barriers to advocacy.

Themes	Cervical cancer (*n* = 11)	Breast cancer (*n* = 10)
**Self-advocacy—facilitators** Knew something was wrong Open with family and friends Relied on a strong support network Trusted their providers Determined to fight Wanted more information Felt connected to others who had cancer Friends and family advocated for them Believes self-advocacy is important **Self-advocacy—barriers** Fear Not opening up to family/friends Not wanting others to worry about them Did not want additional information **Community advocacy—facilitators** Talked with kids about diagnosis Trusted their providers Faith Interest in support groups Desire to help Sees benefit in survivors speaking out **Community advocacy—barriers** Not always feeling supported Felt it could have been worse Struggled during treatment Unclear connection between HPV and cervical cancer Lack of confidence	7 7 9 8 6 7 7 7 4 5 7 4 6 6 8 5 1 5 3 4 6 6 6 5	7 7 10 9 10 6 7 7 5 2 5 6 3 8 9 7 5 7 6 5 5 4 2 3

**Table 4.** Example statements from patients discussing major themes involving facilitators to self-advocacy.

Theme	Patient Quote (CC = cervical, BC = breast)
Knew something was wrong	“Okay. I was having this really bad bleeding and I contacted my gynecologist at the time. It was an emergency; like I felt like, I mean, this is unusual, and I was having these huge clots and I’m like, “Something’s not right.” I was feeling, you know, something was just weird. “ (CC) “And I just had a feeling. Everybody who had felt the lump – acupuncturist, my GP, other practitioners – said that it didn’t feel super alarming; it was movable, it didn’t cause me pain. So, everybody kept trying to like calm me, but I just had a feeling.” (BC)
Open with family and friends	“Well my family’s so close, we can talk to each other about – I mean anything.” (CC) “I immediately called them, and they have been my rock—my go-to.” (BC)
Relied on a strong support network	“They do encourage me a lot even sometimes when I want to give up because I’m in so much pain I just don’t want to do it no more. They’ll say, ‘Mom, you can’t do that. You’ve got other kids. You’ve got grandbabies.’ They try to motivate me the best they can, but it still hurts.” (CC) “You have to go to people and be like, ‘We need help. We cannot do this.’ You can’t do it on—I had to have tons of people come help take care of the kids, be with me during surgery because Chris couldn’t, or be with the kids so that Chris could be with me, drive me to places, help us move. I couldn’t. I just had surgery. I couldn’t pick up anything. Or I had the second surgery, so, I mean. Food, I couldn’t cook. I couldn’t pick up my child.” (BC)
Trusted their providers	“But the doctors here have been totally awesome the whole time…The whole time. They have been there for me every single time.” (CC) “Well, when I first met her, she sat down and she was engaged with me, she listened to me. And over the last four or five years we’ve been coming here, she knows what I need to hear.” (BC)
Determined to fight	“I’m about to kick this thing’s ass. This is not going to ever take me and I’m going to let everybody know.” (CC) "Because I was not going to give up. I refuse to give up. I had so much that I want to do, so much I want to see. I’m still young. And I figured if I could go through the first three rounds of chemo and two rounds of chemo, the radiation on the inside, I said no matter what they throw at me, I’m still going to fight. If I got to fight by myself with me and my doctors, I’m still going." (CC) “So, I knew if I didn’t want to die, I had to keep pushing.” (BC) "I wanted to survive. I wanted to kick this thing." (BC)
Wanted more information	“I wish there was more cervical information out there. Like, you see pamphlets about ovarian cancer all the time, but you do not see that much about cervical cancer.” (CC) “I know some people can have fear from knowing more, but I like to know it.” (BC) “I wish that education was out there. Even in like the [medication] commercial, they’re like, ‘survival-free progression’ or whatever. People do not know what that means.” (BC)
Felt connected to others who had cancer	“My mother-in-law had breast cancer and she would always kind of be like, ‘Nobody understands what I’m going through.’ And then, so, she and I have this bond now that we can kind of – we’re closer because of that.” (CC)
Friends and family advocated for them	“You know, my mom kept telling me I needed to go back to the OB and she’s like, ‘I told you.’ Like I called her and I told her what was going on. She’s like, ‘I told you you were supposed to go back,’ you know. And I’m like, ‘Oh, my gosh.’ You know, I wasn’t paying attention to my body enough or I wasn’t being heard or like I felt like I was being heard. So, there was kind of all those thoughts. And I feel like if it were not for my mom making a call to another OB for me, who knows where I would’ve been after that?” (CC) “Oh, yes, yes, my husband is always my advocate. He pushed things much further than I ever would. He still does. Like he is my advocate still today when I would say yes, I’ll do that. Yes, I’ll go here. He’ll be like, ‘Sorry, she can’t do that. Or if she’s goes with you, you’ve got to watch out for her because she doesn’t know her limits. She still thinks that she has all this energy, or she will push herself to because she wants to be with you, or she wants to get this done.’ So, like he’s still my advocate. I have an amazing husband.” (BC)
Believes self-advocacy is important	“So, I think self-advocacy is so important. And I think there’s still a lot of people, maybe specifically here in the South …and if the doctor says it’s so, then, it is and there’s no question-asking.” (BC) “Who am I going to help if I cannot do anything to help myself? That got my mind going. Boom, I am done with this.” (CC) “And then even for yourself I feel like you need to be able to speak for yourself and like when I tell people you always need to advocate for yourself because if you don’t want to do something than you shouldn’t do it. If you have questions, you need to ask. You need to get answers. You can’t be afraid.” (BC)

When further comparing the patients who expressed a determination to fight in the CC group, all but one identified as an advocate either for themselves or others in their community. Participants in both groups shared that the determination to fight was most often a resolution to live another day. These participants shared a mental strength that was built on past personal experiences or watching others they care for overcome physical challenges. It was directed at their day-to-day survival and strength. They used this internal motivation to ask the questions they wanted to ask, seek second opinions, and reach out for help when needed.

Many of these facilitators revolved around the participant's closest support systems including parents, spouses, children, friends, healthcare providers, neighbors, and other cancer survivors. Many cited gaining the courage to seek care or ask questions regarding their care from family members and friends who encouraged them, accompanied them to appointments, and often started asking the questions or making phone calls for them. When participants fully trusted another person, they were able to draw from their strengths and use it as a reminder of their ultimate treatment goals.

Focusing on the 7 CC and 7 BC participants that identified as self-advocates, 3 from each group were early stage, and 4 from each group advanced stage. Participants of all education levels and all races identified as self-advocates. Their average scores on the FSACS scale were higher in all categories with mean informed decision-making, effecting communication, and connected strength scores of 37.4, 30.9, and 35.6. Their mean total FSACS score was 103.9.

#### Barriers to self-advocacy

Barriers to self-advocacy were reported to be fear, not opening up to family and friends, not wanting others to worry about them, and not wanting additional information. (**Table 5**). Participants reported fear mostly revolved around not knowing what to expect, but also inhibited them from asking questions as they did not want to hear the worst-case scenario. Fear also came as a fear of stigma related to telling the people they cared for. They did not want to be viewed as the person with cancer. Many participants shared concerns about adding stress and anxiety to their family members, and not wanting to speak up or ask for help because they did not want to be a burden to their support system. In contrast to the facilitators revolving around leaning into one’s support system, barriers seem to include isolating oneself. By not involving others or seeking additional information, they felt they could just “go through it and get it done and over with” without identifying with the cancer or being accountable for any outcome. Participants expressed an attempt to disconnect from the cancer experience as a whole.

**Table 5.** Barriers to self-advocacy. Example statements from patients discussing major themes involving barriers to self-advocacy

Theme	Patient quote (CC = cervical, BC = breast)
Fear	"Scared, probably confused, didn’t know what to expect because I’d never had cancer. So, this is a learn as you go type thing. I really didn’t know what to think." (CC) "I was just scared. And it wasn’t like telling them, I was just scared because it had been cut on and ten months, and I was just scared." (BC)
Not opening up to family and friends	“It is embarrassing for them to be leaking everywhere at somebody else’s house, somebody else’s bed, or somebody else’s – in my niece’s bed and stuff like that.” (CC) “I don’t need it to – I don’t want to be known as – I don’t want it to consume me.” (BC)
Not wanting others to worry about them	“They were shocked and sad, worried. Over the time went, I would be so sick. They would just be so concerned and I didn’t want to let them see me depressed, and hurt, and crying, and praying to Lord.” (CC) “She don’t want no pity party. She don’t want nobody feeling sorry for her, so she just don’t worry about anything. She just go.” (BC)
Did not want additional information	“I’ve read up on stuff that they gave me. But I would stop reading because the fear would get into me. You know what I’m saying? So, I didn’t want to read anymore. I didn’t want to hear anymore. So, it was just me thinking—me not knowing is better for me. So that’s how it’s been so” (CC)

CC participants were more likely to report fear, not opening up to family, and not wanting additional information. They reported insecurities related to ostomies and fistulas, unique to the CC group, as additional reasons to withdraw from their support systems. The potential role of a stigma against HPV is a possible contributor to these feelings and will be discussed in greater detail in reference to community advocacy.

#### Facilitators of community advocacy

Talking with their kids about the diagnosis, trusting their providers, strong faith, interest in support groups, desire to help, and seeing a benefit in survivors speaking out were all found to be facilitators of community advocacy. Similar to self-advocacy facilitators, many of these revolved around feeling supported and encouraged by those around them (**Table 6).**

**Table 6.** Facilitators to advocacy in the community. Example statements from patients discussing major themes involving facilitators to community advocacy.

Theme	Patient Quote (CC = cervical, BC = breast)
Talked with kids about diagnosis	“And I said well, when do we tell the girls or what do I tell the girls. And he was like well, you tell the truth. You tell them everything you know. And then my girls had a thousand questions because they are two teenage girls. And so, I told them everything. And they had a thousand questions.” (CC) “I worry about my children. I worry a lot about them----because they don’t understand. They’re 12 and 10 now. It’s hard to explain to that child why I feel bad and can’t do the things that all their friends families are doing because I can’t get out of bed. That’s been hard.” (BC)
Trusted their providers	“But the doctors here have been totally awesome the whole time…The whole time. They have been there for me every single time” (CC) “Well, when I first met her, she sat down and she was engaged with me, she listened to me. And over the last four or five years we’ve been coming here, she knows what I need to hear.” (BC)
Faith	“Of course, I do believe in the good Lord. So, I know he’s doing this for a reason. I just don’t know why. But it’s not my place to ask him that. Yeah.” (CC) “So, I tell myself all the time, I say, well, God gave me another opportunity at life.” (BC)
Interest in support groups	“So, as soon as we got here, I just put out a feeler on Facebook to see if there were any local breast cancer survivors and two women responded. So, we met for the first time maybe in May. And I still run the group; we meet monthly.” (BC) “But having to talk to other survivors is very helpful too because they can say okay, well, you’re going to be going to this, this is what you need to look for, this is what you need to start doing now. Start, make sure you drink plenty of water, exercise, call us if you need help. We’re going to do things. We’re going to support each other. You know, it’s just good to have someone that’s actually been through it because even your family, you can’t really tell them like everything you feel.” (BC)
Desire to help	“I am like if someone else that I know that has it ever popped up, I was like hell yeah, I would be the first one on there going hey. Need me to go to the appointment? I am there. Let us go. I had a few people ask me. Hey, do you want me to come to your appointment.” (CC) “I felt – I’m the type that I like to give back because people have given to me for years. And just like even with [doctor], she – I don’t know, I feel like she has given to me and I feel like if there’s any way I can help anyone.” (BC)
Sees a benefit in survivors speaking out	“Because I’ve been, somebody else needs to know it’s not the end.” (CC) “I think I think it was very healing for people and so I always encourage people to tell their story.” (BC)

Telling their children about a cancer diagnosis seemed to open a door not only for internal motivation to continue treatments and maintain a positive attitude but also an avenue to begin to talk to more people in their social circle as well. Both breast and cervical cancer have excellent screening tools that many participants expressed stressing to their children. They sought to educate their own families about appropriate prevention and screening strategies in an effort to protect them in the future.

BC participants were more likely to express an upfront interest in support groups and see a benefit in survivors speaking out. They mention immediately turning to social media or others they know who have experienced cancer to seek advice and support upon receipt of their diagnosis. While the difference in the incidence of these diseases and therefore the availability of these resources likely plays a role in this noted discrepancy, CC participants also reported a lack of local groups, fear of negative opinions, and an unawareness of cervical cancer support groups as reasons to not participate.

When asked to describe what advocacy is, most participants discussed things associated with advocacy in the community, speaking out about their experience, supporting someone going through a similar challenge, and providing education. Participants who reported participating in community advocacy reported sharing testimonials at events at their cancer centers and churches, planning fundraisers at schools, posting on social media, writing a book, talking with various groups in their communities, and acting as a companion to doctor appointments for others in their community.

Focusing on the 7 CC and 6 BC participants who identified as community advocates 5 CC patients were early stage while only 2 were advanced stage. The BC participants were split evenly with 3 being early stage and 3 being advanced. Participants of all education levels and all races were included in this subgroup. They had a higher average total FSACS score of 105.1 compared to the total cohort average score of 100.9.

#### Barriers to community advocacy

Major barriers to community advocacy were not feeling supported, feeling they could have had it worse, struggling during treatment, fear, a lack of confidence, and having an unclear connection between HPV and cervical cancer (**Table 7**). When discussing a lack of support many participants brought up feeling that others don’t understand what they are or have gone through. They feel their friends and family want them to be back to who they were before they were diagnosed with cancer but the challenges they went through do not disappear as soon as their treatment is completed. Because they feel unsupported, they often limit their activities outside the home which in turn limits their opportunities for advocacy in their communities.

**Table 7.** Barriers to advocacy in the community, Example statements from patients discussing major themes involving barriers to community advocacy.

Theme	Patient quote (CC = cervical, BC = breast)
**Not always feeling supported**	“No, because I don’t have friends. And I don’t have friends. Because, I mean, do you know what the – what people are like? No, I’ve never had, and I’ve never told people about my life, either. Nobody. I’ve never had a – a close friend.” (CC) “What’s hard is that I have siblings that they don’t want to accept where I am right now. They want me to be well and isn’t that crazy that your family will ostracize you because they don’t know what to do with it?” (BC)
**Felt it could have been worse**	“Because I did not fight half as hard as these women that fight for it or fight with it. “(CC) “And then the joint pain and all that, so I feel like I’ve aged really fast the past few years. But but then I, I, like, again I, I knew I could say, ‘Well, it could have been worse,’ that kind of thing.” (BC)
**Struggled during treatment**	“I’ve been plagued with physical issues since treatment and I can’t work anymore. So, now I’m going to lose my train of thought what I was about to say because that’s one of my symptoms. My mind goes out on me.” (BC) “All these holes. It is so hard to get used to. I wake up, and I am like, all the bags are full. This one is full. When I would get up, this one would bust. You are at somebody else’s house, and it is embarrassing. That is the most hard thing for me. The cancer itself does not bother me.” (CC)
**Unclear connection between HPV and cervical cancer**	“When that tumor was doing everything it was doing and smelling horrible, all the sewer water was coming. It was just sewers. That is what it smelled like. I have never in my life. I am 40-something years old, I never had any type of venereal disease, nothing like that. I did not know what it was. I could have caught something from the toilet seats at work. I did not know. I did not know. Yeah, they give you a little whatever home health and healthcare in school or whatever. Boils, whatever the hell gonorrhea crap, and the oozing.”(CC) “I don’t even know if I understand what those two’s got to do with each other. I know it’s related, I think. But nobody’s actually sit down and explained it to me. If they did, I done forgot already.” (CC)
**Lack of confidence**	“I probably need to gain confidence in me and be happy with me. It’s so hard to be happy about yourself when you don’t know your purpose, when you don’t know what you’re good at, when you don’t know.” (CC) “I think it takes someone who has got the knowledge, number one, to build it to understand more of what I am… I do not know everything. So, to have more knowledge about cervical cancer than I have.” (CC)

A large part of survivor advocacy involves being vulnerable and willing to share your own experience. These patients commonly cited a lack of confidence, not being “really good at that” or being “kind of shy” as reasons to not speak up at events or to groups of people. Some expressed feeling a lack of validity in their experience since others may have had a harder course, a more advanced stage, or more complications. Despite having completed cancer therapy, they felt they may not be the most qualified to share their experience.

When discussing the effect of the association of HPV with cervical cancer on participant’s feelings towards speaking up about their experience, 6/11 of cervical patients were unaware of the association between HPV infection and cervical cancer. Comparatively, 2/10 of breast cancer patients were unaware of the connection. A CC participant expressed, “I don’t even know if I understand what those two’s got to do with each other. I know it’s related, I think. But nobody’s actually sit down and explained it to me. If they did, I done forgot already.” The significant disparity in understanding the root cause of cervical cancer likely led to the lack of perceived stigma against STDs being reported as a barrier. A lack of clear understanding of the disease process directly inhibits those survivors from educating the community on screening and prevention.

## Discussion

Although BC survivors are classically thought to be more involved in advocacy, this study suggests that both BC and CC patients participate in self-advocacy and advocacy behaviors. Additionally, they share many similar drivers to being advocates.

While detailed facilitators for self-advocacy and community advocacy differed, many revolved around the larger concept of having a strong support network. Opening up to family and friends, relying on a strong support system, trusting their providers, feeling connected to others who had cancer, having family and friends advocate for them, talking with kids about their diagnosis, and having an interest in support groups all involve positive interactions with other people in their lives. Many of the barriers discussed are also related to not having strong relationships or withdrawing away from some. Every participant mentioned relying on someone else at some point in their journey, whether it be a family member, doctor, nurse, or neighbor. These individuals played a variety of roles. They were a source of encouragement to speak up for themselves, the first person to whom they could openly express their own experience, and the first person to like a social media post. Numerous studies and meta-analyses have found higher levels of social support to be associated with higher health-related quality of life, decreasing pain scores, and decreasing risk of mortality.^15-17^ A large meta-analysis of 148 studies found a 50% decrease in the risk of mortality in patients with stronger support networks. A study looking specifically at patients with breast cancer found social isolation to be associated with a higher risk of all-cause mortality. Additionally, the quality of the support, measured by the amount of time spent with people in their circle or doing things with others, was more protective than the size of the social network.^18^ Many of our participants who reported advocacy behaviors reported having small interpersonal communities but would frequently mention one or two people who made an impact on their treatment and survivorship journeys. As the healthcare system continues to decrease the length of hospital stays and move towards more in-home care, caregivers and support people are playing a larger role in the treatment of cancer patients. With the potential impact on both quality of life and mortality, it is imperative that the healthcare system begins to integrate these individuals into the care they provide.

The other overwhelming concept discussed was discomfort with themselves or their experience. Feelings of fear, lack of confidence, invalidity of their course, and lack of understanding were continually expressed as barriers to advocacy behaviors. Six CC patients and two BC participants stated that they did not know that there was an association between cervical cancer and HPV. HPV is surrounded by stigma associated with sexually transmitted infections and patient guilt regarding inadequate screening.^19-21^ However, a stigma against HPV was not something commonly discussed by these participants, even when prompted about HPV in the topic guide. There is no data to suggest this stigma persists outside of patients with cervical dysplasia and in this patient population, a lack of education and understanding likely contributed to a lack of perceived stigma around HPV. This finding highlights the desperate need for additional education, both in our communities and among our cancer survivors. More than 60% of all participants in each group reported a desire for additional information during treatment and following treatment completion. Improved access to education was also frequently given as a recommendation for ways to improve care. Efforts need to be made to increase education in patient-friendly language.

Some patients suggested a visit between the initial diagnosis and treatment initiation for additional education and questions. Another study suggested waiting until treatment completion, when the patient may be more prepared to absorb information. Following the Institute of Medicine, which highlighted the need for more comprehensive care of cancer survivors, survivorship clinics have gained popularity around the country and have been shown to increase patient education and understanding, decrease distress, and improve the severity of chronic symptoms.^22-25^ Despite these benefits and the reports of success of survivorship clinics for breast, head and neck, and blood cancers, to our knowledge, there has not been any published data regarding focused survivorship clinics for patients with gynecologic malignancies. This offers another common difference in the experiences of BC and CC patients, and an additional opportunity to improve the care, education, and quality of life of patients with cervical cancer.

While the majority of patients in each group reported being an advocate in the community or an advocate for themselves, only four participants in each group reported being both self-advocates and community advocates. The facilitators and barriers for both self-advocacy and community advocacy had many similarities and overarching concepts, However, there remains a discrepancy among patients regarding how they perceive themselves, and the behaviors they choose. There were no cohesive themes expressed more by those who identified as self-advocates versus community advocates. In fact, both groups expressed similar themes. Further studies are needed to evaluate potential personality traits that may impact this finding.

Many factors should be considered when discussing the strengths and limitations of this study. To our knowledge, this is the first study to evaluate advocacy efforts within a population of patients with cervical cancer. This study also took a unique approach to clearly identify what personal and social factors may contribute to a patient’s desire or ability to be an advocate, either for themselves or for others. Although we were able to reach saturation in this study, our sample size was still relatively low. Given the interview style of the study, there is potential for selection bias, as patients who were willing to participate in the study may be more outspoken and willing to discuss their disease process than those who chose not to participate.

## Conclusions

Cervical cancer survivors have been reported to have some of the lowest quality of life (QOL) scores compared to other gynecologic and non-gynecologic malignancies.^26,27^ Data supports the role of advocacy behaviors in improving cancer survivors’ QOL.^1-3,5^ Having a strong support system and feeling a desire to fight or help are powerful facilitators to both self-advocacy and community advocacy efforts. Ultimately, the information gathered can be used to create systems change and provide interventions that encourage strengthening social networks and improving education both during treatment and after.

## Supplementary Material

oyaf130_suppl_Supplementary_Material

oyaf130_suppl_Supplementary_Tables_S1

## Data Availability

Data relevant to the current study are available from the corresponding author upon reasonable request.
