# Supplementary material for: Supporting our survivors: an evaluation of the facilitators and barriers to advocacy in cervical and breast cancer survivors
Source: Oncologist. 2025 Jul 22;30(7):oyaf130. doi: 10.1093/oncolo/oyaf130 (PMC12362239; doi:10.1093/oncolo/oyaf130)
Supplement: oyaf130_suppl_Supplementary_Material [file oyaf130_suppl_supplementary_material.docx]

**Supplement 1: Topic Guide**

Tell me how you felt when you were first diagnosed with cancer. Identify initial feelings of fear, guilt, or shame they felt with their diagnosis. What was their understanding of their disease and causes of their disease.

Who did you tell about your diagnosis? Did they share news of their diagnosis with their family?

Where you ever embarrassed by your diagnosis? Assess for any stigma they feel towards their cancer diagnosis.

What was your greatest fear at that time? Did they fear treatment, shame from their family/friends, death?

Where did you get information regarding your disease? Did you feel as though you had resources?

Looking back, what has been the most challenging part of having cancer?

What strengths of yours do you think helped you get through this time?

Do you feel like you have had adequate support from others during that time? Explore support from family, friends, healthcare providers.

Do you feel that your personal relationships changed as a result of your diagnosis? Did people distance themselves, were they able to find strength in their relationships?

Can you describe your relationship with your healthcare providers? Explore their relationship with physicians, nurses, techs.

How would you prepare for your appointments? List of questions, bring family, etc.

Did you feel as though you were able to receive help in between appointments if needed? Did they have access to the on-call line, emergency room, online portal?

**Now I want to talk at little about advocacy.**

When I mention “advocacy,” what does that mean to you?

What do you envision when you picture an advocate? What about an advocate for yourself?

Who would you describe as an advocate in your life?

Do you feel that you are an advocate? Explore feelings of self-advocacy or advocacy within their social circle or greater community.

What would you say to patients who have been recently diagnosed with cervical/breast cancer?

What would you say to patients who are not up to date on screening?

What would you say to patients who are scared to follow up after an abnormal screen?

Would you ever be interested in being involved in advocacy efforts in the community? (encouraging patients to get screened, vaccinated, following up, genetic testing, etc.)

Would you ever or have you ever spoken out publicly about your experience?

What would make you feel more comfortable speaking out about your experience?

What things could healthcare providers do to encourage you to be more outspoken about your experience?

Do you have any ideas for ways to improve screening or follow up in the community?
