# Supplementary material for: Supporting our survivors: an evaluation of the facilitators and barriers to advocacy in cervical and breast cancer survivors
Source: Oncologist. 2025 Jul 22;30(7):oyaf130. doi: 10.1093/oncolo/oyaf130 (PMC12362239; doi:10.1093/oncolo/oyaf130)
Supplement: oyaf130_suppl_Supplementary_Tables_S1 [file oyaf130_suppl_supplementary_tables_s1.docx]

**Supplemental Table S1. Staging Categorization**

|  | **Cervical Cancer** | **Breast Cancer** |
| --- | --- | --- |
| **Early Stage** | Stage IA1-IB2 | Stage I-IIIA |
| **Advanced Stage** | Stage IB3 - IVB | Stage IIIB-IVB |
